# Design of a Novel Trabecular Acetabular Cup and Selective Laser Melting Fabrication

**DOI:** 10.3390/ma15176142

**Published:** 2022-09-04

**Authors:** Congyu Wang, Baoyu Sun, Yongdi Zhang, Congwei Wang, Guang Yang

**Affiliations:** College of Mechanical Engineering, Hebei University of Science and Technology, Shijiazhuang 050018, China

**Keywords:** acetabular cup, porous structure, gradient porosity, Ti6Al4V alloy, selective laser melting

## Abstract

The acetabular cups used in total hip arthroplasty are mostly made of dense metal materials with an elastic moduli much higher than that of human bone. This leads to stress shielding after implantation, which may cause aseptic loosening of the implant. Selective laser melting (SLM) technology allows us to produce tiny and complex porous structures and to reduce the elastic moduli of dense metals, thereby avoiding stress shielding. In the present study, rhombic dodecahedron porous structures with cell sizes of 1 mm, 1.5 mm, and 2 mm were designed. The strut diameter was changed to ensure that the porosity and pore size would meet the bone ingrowth requirements. Then, porous Ti6Al4V alloy specimens were printed using SLM, and compressive tests were carried out. The results showed that the compressive strength and elastic modulus values of the specimens with a cell size of 1.5 mm were in the range of 78.16–242.94 MPa and 1.74–4.17 GPa, respectively, which are in line with the mechanical properties of human cortical bone. Finite element analysis of a total hip joint model was carried out to simulate gait, and the surface of the trabecular acetabular cup was divided into 10 regions according to the stress distribution, with the stress interval in the range of 37.44–219.24 MPa. According to the compression test results, the gradient structure of Ti6Al4V alloy with different porosity was designed for trabecular coating. The gradient porous structure meets the mechanical requirements and is closer to the natural structure of human bone than the uniformly distributed porous structure.

## 1. Introduction

The hip joint is one of the largest joints in the human body, and it enables many complex body motions in our daily life. However, the hip joint is prone to various types of diseases, such as femoral head necrosis and osteoarthritis. Once such diseases affect daily activities, it is necessary to consider replacing the hip joint with a prosthesis through total hip arthroplasty [1]. The acetabular cup is one component of a hip prosthesis. Acetabular cups are typically made of dense metal by traditional machining methods. However, the elastic modulus of dense metal (Ti6Al4V, 113 GPa; 316 L stainless steel, 210 GPa; and CoCrMo alloy, 240 GPa) is much higher than that of human bone (0.02–20 GPa) [2]. As a result, there is often stress shielding between the implant and the bone, meaning the implant is subject to more load than the surrounding bone [3]. According to Wolff’s law, there is a state of physiological balance between bone tissue and any loads exerted on it. Stress shielding can easily lead to bone resorption, cortical bone thinning, and eventually implant loosening [4]. In addition, the dense metal structure prevents bone tissue from growing in and, thus, does not provide long-term stability after implantation, which is an important cause of loosening.

Porous structures are interconnected networks composed of solid rods or plates that form the edges and walls [5]. Weevils, beetle shells, and butterfly wings are examples of porous structures found in nature [6]. Porous structures have the characteristics of low relative density, large specific surface area, high specific strength, and good energy absorption, so they have great potential for application in various fields [7]. For example, they have been used in the medical field to create implants imitating the structure [8] and elastic modulus of human bone to reduce stress shielding. In addition, pores provide conditions for cell adhesion, diffusion, and differentiation, and the interconnected structure facilitates the circulation of oxygen and nutrients, thereby promoting bone growth [9].

Porous materials can be fabricated using traditional methods, such as powder metallurgy [10] and the material foaming method [11]. However, with these methods, the pore size is usually small and non-uniform, and the through-hole ratio is low. Hence, such materials do not meet the needs of medical applications [12]. Selective laser melting (SLM), which is one approach to 3D printing, is a transformative manufacturing technology developed in recent years. Based on a digital model and adopting the material layer-by-layer accumulation method to manufacture solid parts [13], SLM selectively irradiates the surface of metal powder with a high-energy laser to make it rapidly fuse. The desired three-dimensional model is obtained through layer-by-layer accumulation [14,15]. Compared with traditional manufacturing techniques, SLM can realize the free forming of porous structures with very high precision [16], and it has the advantages of fast forming speed, good mechanical strength of formed parts, and high material efficiency [17,18,19].

The main goal of this study is to design and optimize the porous layer on the outer surface of the traditional titanium alloy acetabular cup, so that it not only meets the mechanical strength demand of the hip joint during normal walking, but also alleviates the “stress shielding” effect to the greatest extent. Through changing the porosity and cell size of the porous structure, etc., porous titanium alloys with different mechanical properties can be obtained [20]. Therefore, some porous Ti6Al4V alloy specimens were printed by SLM technology to obtain the optimal cell size of porous structure through compression tests. The mechanical analysis of the acetabular cup was carried out in the complete gait cycle, and the porosity of each region of the porous layer was determined based on the stress distribution of the acetabular cup, so a porous layer with gradient porosity was formed. The acetabular cup was designed based on the cyclic loading of human gait, its mechanical properties were similar to human bone and pore size, and porosity was superior to existing acetabular cups. The gradient structure of the new acetabular cup could better match the mechanical properties of human bone and improve patient comfort.

## 2. Materials and Methods

### 2.1. Materials

**Figure 1:**
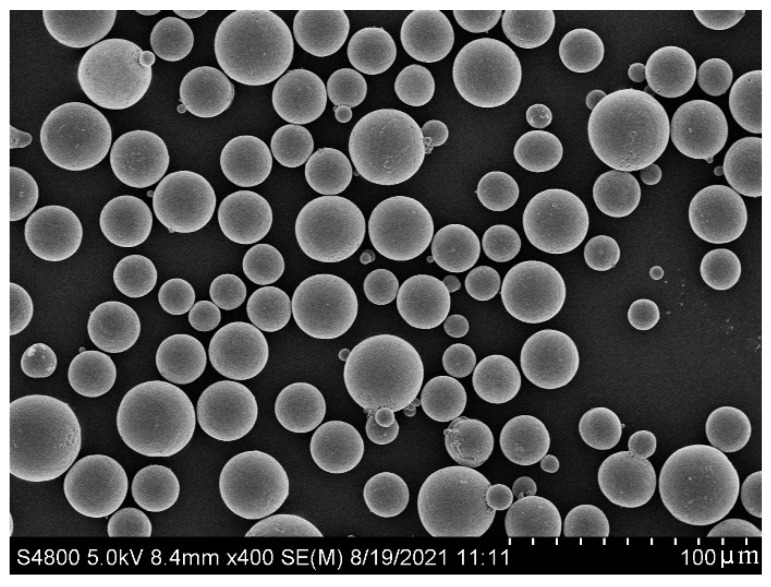
Microscopic morphology of Ti6Al4V ELI alloy powder.

The Ti6Al4V alloy is a common material used for orthopedic prostheses due to its good mechanical properties, biocompatibility, and corrosion resistance [21,22,23], and new titanium alloy materials have been continuously developed [24,25,26]. The Ti6Al4V alloy is one of the most commonly used titanium alloys in medical field. Therefore, in this study, Ti6Al4V ELI powder (AP&C, Saint-Eustache, QC, Canada) was used for SLM. The chemical composition of the powder, shown in Table 1, meets the requirements of ASTM F136. Figure 1 shows the microscopic morphology of the powder. Less than 1.5% of the particles have a sphericity lower than 0.8. The powder particle size is D10 = 23.0 μm, D50 = 32.5 μm, and D90 = 45.4 μm. The bulk density is 2.5 g/cm^3^. The tapped density is 2.82 g/cm^3^. The Hall flow rate is 38.6 s/50 g. Thus, it has good powder fluidity and can provide a good powder spreading effect during the molding process.

### 2.2. Methods

**Figure 2:**
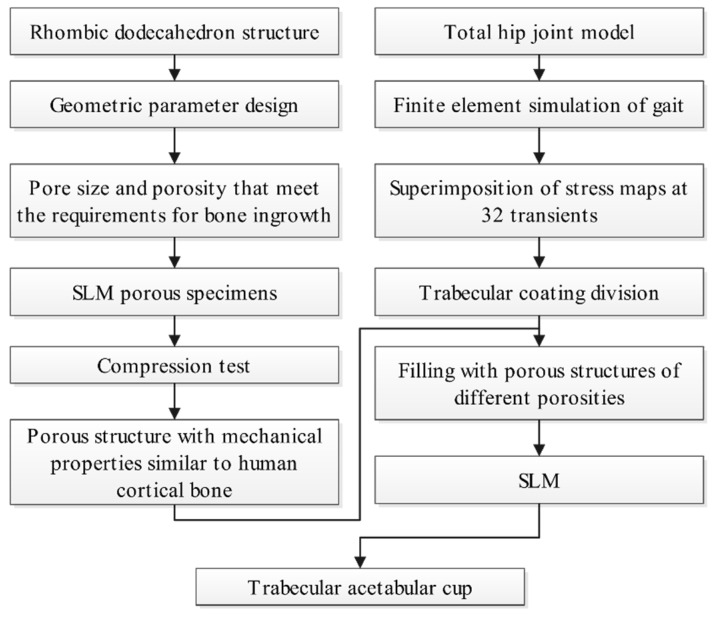
Flowchart of design and SLM fabrication of the trabecular acetabular cup.

In this study, we proposed a new design for a trabecular acetabular cup with a gradient porous structure, which can be fabricated based on SLM technology. The porosity of the porous structure was varied across different regions of the cup surface according to the stress distribution on the cup. First, a porous structure that was suitable for bone ingrowth was designed, compression specimens were fabricated using SLM technology, and the compression performance was tested in order to obtain a porous structure with mechanical properties similar to that of human cortical bone. Then, finite element analysis of the total hip prosthesis was performed under gait conditions to obtain the stress distribution of the trabecular coating on the surface of the acetabular cup. Based on the mechanical properties of the porous structure, the porosity of the trabecular coating was changed. Lastly, SLM technology was used to form a prototype. Figure 2 shows the technical route of this study.

**Figure 3:**
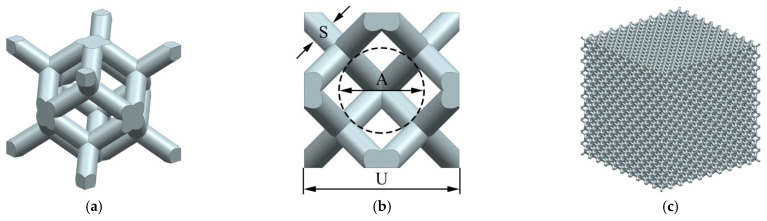
Rhombic dodecahedron porous structure: (**a**) geometry; (**b**) geometric parameters; (**c**) specimen model.

A rhombic dodecahedron porous structure was designed using the 3D modeling software UG 12.0 (Siemens PLM Software, Plano, TX, USA), as shown in Figure 3a. The rhombic dodecahedron has four triple symmetry axes, three quadruple symmetry axes, and nine symmetry planes, which can remain stable under multi-directional pressure. Therefore, it is an ideal porous structure for implants. The geometric parameters of the porous structure include the cell size U, the strut diameter S, and the pore diameter A, which is the diameter of the largest inscribed circle of the porous structure, as shown in Figure 3b. The porosity *P* of the porous structure is the ratio of the pore volume to the volume of the specimen, which can be calculated by the following equation:

(1) $$P=(1-\frac{V}{V_{S}})\times 100\%$$

where *V* is the volume of the specimen, and *V_S_* is the volume of the largest peripheral boundary of the specimen. Porous structures with three different cell sizes, namely 1 mm, 1.5 mm, and 2 mm, were designed. By changing the strut diameter, the porosity was controlled at 60–90%, and the pore size was 500–1200 μm (Table 2), which meets the requirements for bone ingrowth [27,28]. The specimen was composed of 10 × 10 × 10 cells in three directions, as shown in Figure 3c.

**Figure 4:**
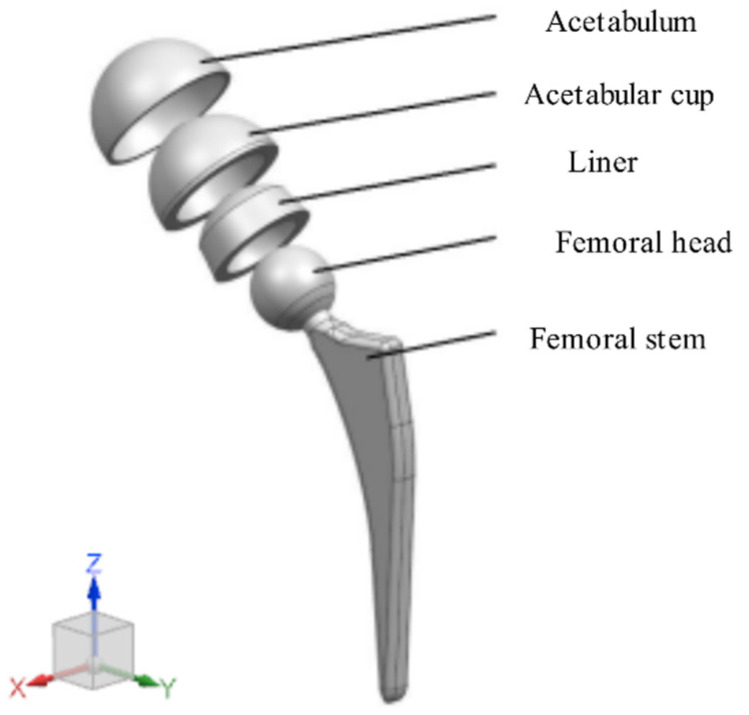
Finite element model of the total hip joint.

**Figure 5:**
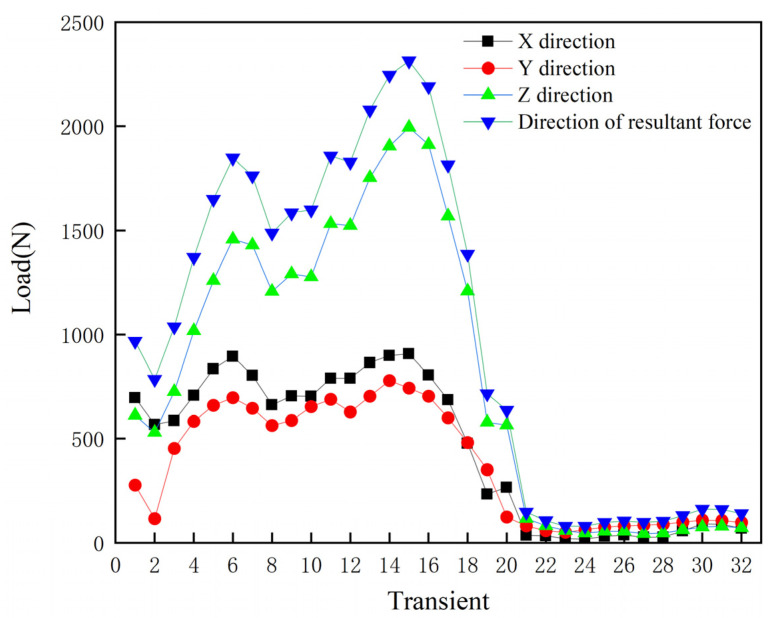
Joint loads at 32 transients of the gait cycle.

A hip prosthesis model was created using UG 12.0 software, which mainly included the acetabulum, acetabular cup, liner, femoral head, and femoral stem (Figure 4). In order to simulate the state of the acetabular cup installed in the acetabulum and analyze the force of the acetabular cup during gait movement, according to the research of Mircheski et al. [29], a hemispherical shell with a thickness of 2 mm was established on the outer surface of the acetabular cup to simulate the contact cortical bone. The model was imported into finite element analysis software ANSYS Workbench 2019 R3 (ANSYS, Canonsburg, PA, USA). The material properties are shown in Table 3 [30,31]. In order to simulate the actual installation of the acetabular cup, the acetabulum is set to fixed, the contact relationship between the acetabulum and the acetabular cup is set to bonded, the contact relationship between the liner and the inner wall of the acetabular cup and the femoral head is set to frictional, and the friction coefficient is 0.1. The model was meshed with 1 mm tetrahedral elements, the total number of elements was 604,487, and the total number of nodes was 951,848. Pedersen et al. [32] divided a human gait cycle into 32 transients and measured the forces in the X, Y, and Z directions on the hip joint at each transient (Figure 5). In this study, the gait data measured by Pedersen et al. were used as the load conditions of the prosthesis model.

The porous Ti6Al4V specimens and trabecular acetabular cup were printed using a Concept Laser M2 (Concept Laser, Lichtenfels, Germany) 3D printer. The printing parameters are shown in Table 4. The parameters were obtained by orthogonal test, and the density of the formed Ti6Al4V alloy was above 99.5%. During the printing process, 99.99% pure argon was used as the protective gas to keep the oxygen content in the chamber below 1000 ppm. The microstructure of the powder was observed using a Hitachi S-4800 (Hitachi, Tokyo, Japan) field emission scanning electron microscope (with an acceleration voltage of 5 kV for SEM). The morphologies of the struts and pores of the specimens were observed with an Optiv Classic 322 (Hexagon, Stockholm, Sweden) measuring machine. The device adopts a glass grating system with a resolution of 0.5 μm, and its X, Y, and Z axis travel ranges are 300, 200, and 200 mm, respectively. It is equipped with a coaxial zoom lens and has the advantages of non-contact and magnification measurements. A CMT5105 (MTS, Eden Prairie, MN, USA) electronic universal testing machine was used to test the compression properties of the specimens. The compression speed was set to 1 mm/min. Each type of specimen was tested three times, and the average results were calculated for analysis.

## 3. Results and Discussion

### 3.1. Compression Properties of Porous Ti6Al4V Alloy

**Figure 6:**
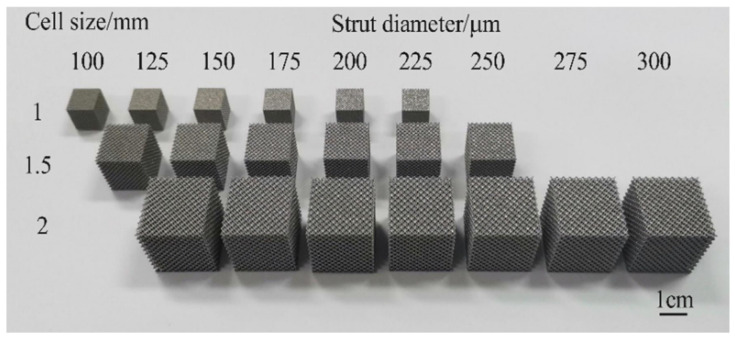
SLM porous Ti6Al4V alloy compression specimens.

**Figure 7:**
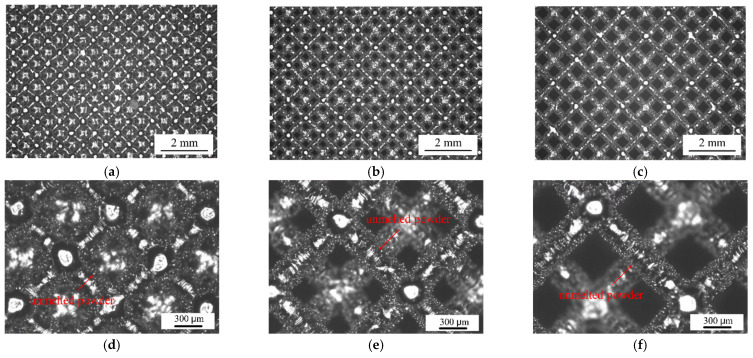
Morphology of the struts and pores of SLM porous Ti6Al4V specimens: (**a**) cell size 1 mm; (**b**) cell size 1.5 mm; (**c**) cell size 2 mm; (**d**) cell size 1 mm; (**e**) cell size 1.5 mm; (**f**) cell size 2 mm.

After formed, the specimens were sandblasted, and then were removed from the substrate by wire cutting. Finally, the specimens were cleaned in an ultrasonic cleaning device equipped with anhydrous ethanol. Figure 6 shows the SLM porous Ti6Al4V alloy compression specimens, and Figure 7 shows the morphologies of the struts and pores of the specimens with cell sizes of 1, 1.5, and 2 mm and the strut diameter of 150 μm. The printed specimens had good morphologies, and there was some unmelted powder on the surface of the strut because of the molten pool formed during SLM. There is a heat-affected zone at the edge of the molten pool, and the temperature of the heat-affected zone is lower than the temperature of the molten pool, so the powder was not completely melted and bonded to the strut surface after cooling (Figure 7d–f).

**Figure 8:**
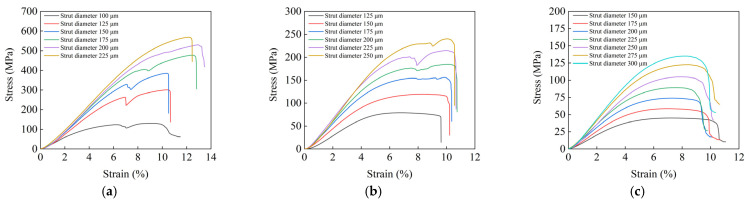
Stress–strain curve of the compression test: (**a**) cell size 1 mm; (**b**) cell size 1.5 mm; (**c**) cell size 2 mm.

Figure 8 shows the stress–strain curve of the specimens in the compression tests. From Figure 8, it can be seen that the specimens first underwent linear elastic deformation under the applied load, and the curve was a straight line with a certain slope. As the load reached the linear elastic limit of the specimens, the slope of the curve changed and the stress dropped off abruptly after reaching the peak, indicating yielding of the specimens. According to previous studies, the compressive strength of the specimen is the first peak stress on the curve [33], and the elastic modulus is the slope of the straight line in the linear elastic stage.

**Figure 9:**
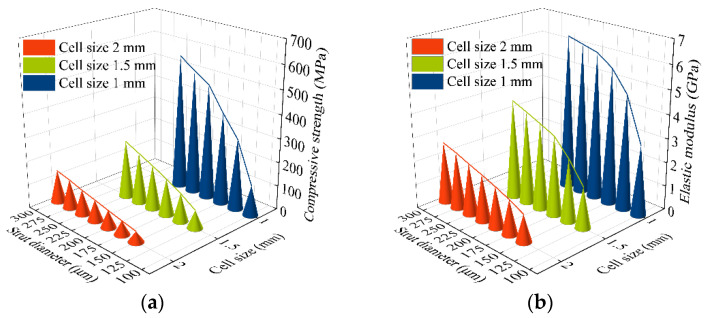
Mechanical properties of porous Ti6Al4V alloy: (**a**) compressive strength; (**b**) elastic modulus.

The variation of the compressive strength and elastic modulus of each specimen is shown in Figure 9. When the cell size was the same, the compressive strength increased with the increase in the diameter of the strut. When the diameter of the strut was the same, the compressive strength decreased with the increase in cell size. This is because, when the cell size of the specimen is the same, the relative density increases with the increase in the diameter of the strut; hence, the compressive strength is increased. When the diameter of the strut remains the same, the length of the strut increases with the cell size, and the growth rate of the pore volume is greater than that of the cell volume, which will increase the porosity; hence, buckling is more likely to occur during compression, i.e., the compressive strength is reduced. This is consistent with the trend of published results [34,35]. The compressive strength of human cortical bone is 100–230 MPa and the elastic modulus is 2–20 GPa [36]. Porous specimens with cell sizes of 1, 1.5, and 2 mm and different strut diameters were tested. Three replicate specimens of each type were tested, and the measurements were averaged. The specimens with a cell size of 1.5 mm have a compressive strength of 78.16–242.94 MPa and an elastic modulus of 1.74–4.17 GPa, which are close to the values of human cortical bone. Thus, 1.5 mm was selected as the cell size for the porous structure used to fill the trabecular coating of the acetabular cup.

### 3.2. Finite Element Simulation of the Acetabular Cup under Gait Conditions

**Figure 10:**
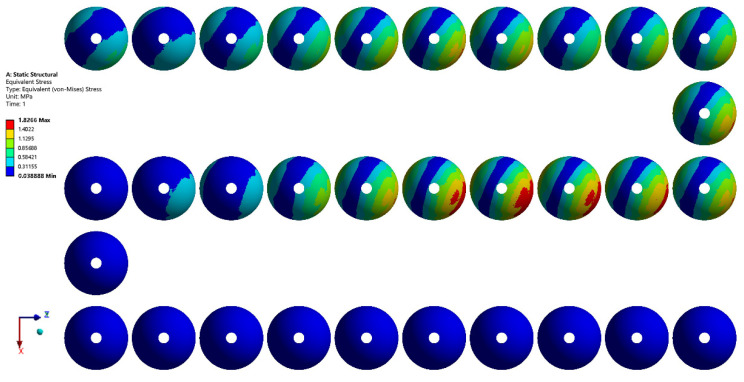
Stress map of the trabecular coating on the surface of the acetabular cup at 32 transients of the gait cycle.

Finite element simulation of the hip prosthesis model under gait conditions was performed using the ANSYS Workbench software, and the stress map of the trabecular coating of the acetabular cup was obtained. The scale of the stress results in each transient was different. For the purpose of comparison between different transients, each stress map was adjusted to the same scale, as shown in Figure 10. The area with the same color in the stress maps of different transients represents the same stress interval.

**Figure 11:**
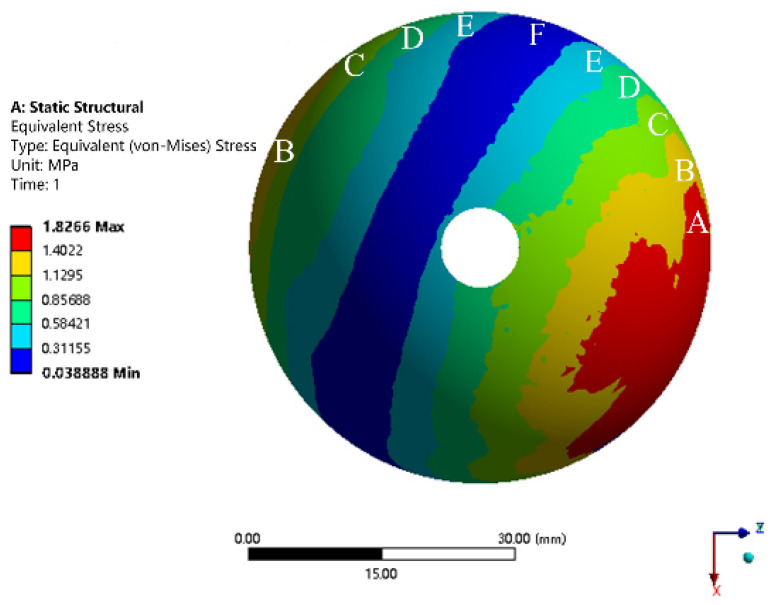
Superimposition of stress map at each transient.

It can be seen from Figure 10 that, since the load during walking is mainly in the Z direction, the stress distribution map is not symmetrical with respect to the Y axis. Since the loads in the X, Y, and Z directions are constantly changing during walking, the stress of the trabecular coating and its distribution are also changing. Transients No. 1 through No. 20 corresponded to the stance phase of the gait cycle. During this phase, the stress peaked at transient No. 15, and the stress distribution was gradient. Transients No. 21 through No. 32 corresponded to the swing phase of the gait cycle. The stress of the trabecular bone was generally small. The stress maps at all transients were then superimposed (Figure 11). The trabecular coating can be divided into 10 regions according to 6 stress intervals from A to F.

### 3.3. Design of Trabecular Acetabular Cup and Selective Laser Melting Fabrication

According to the results of the finite element analysis, the trabecular coating was segmented into 10 regions in the UG software. Since the compressive strength of human cortical bone is 100–230 MPa, that is, the stress of the hip joint will not be higher than 230 MPa during daily activities, the compressive strength of porous Ti6Al4V alloy needs to be just lower than 230 MPa. The stress of the trabecular coating of the acetabular cup was in the range of 0.31–1.83 MPa. Based on the maximum stress of 1.83 MPa of the trabecular coating and the maximum compressive strength of 230 MPa of cortical bone, the safety factor of the trabecular coating was set to 120. The stress value in each region was scaled to a value close to the compressive strength of cortical bone. In this way, while ensuring the strength and safety of the trabecular coating, the compressive strength of the trabecular coating was also close to that of cortical bone. As a result, the mechanical properties of cortical bone and the prosthesis were comparable. According to the mechanical properties of the porous Ti6Al4V alloy with a cell size of 1.5 mm, the trabecular coatings were filled with porous structures with corresponding compressive strengths, as shown in Table 5.

**Figure 12:**
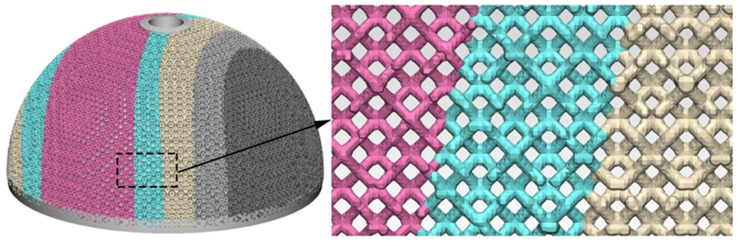
Trabecular acetabular cup model.

The acetabular cup model was imported into the medical modeling software Materialise 3-Matic. Different regions of the trabecular coating were filled with the rhombic dodecahedral porous structure with corresponding porosity, and the trabecular acetabular cup model was obtained (Figure 12). The connection between two adjacent regions with different porosity was excellent. The proposed method of changing the porosity of the trabecular coating of the cup according to the stress distribution of the trabecular coating increases the porosity of the low stress region while ensuring the coating possesses the necessary mechanical properties. The increased porosity will not only provide space for bone ingrowth [37], thereby ensuring the stability of the implant after implantation, but also realizes the lightweight design of the acetabular cup, thereby reducing unnecessary material waste.

**Figure 13:**
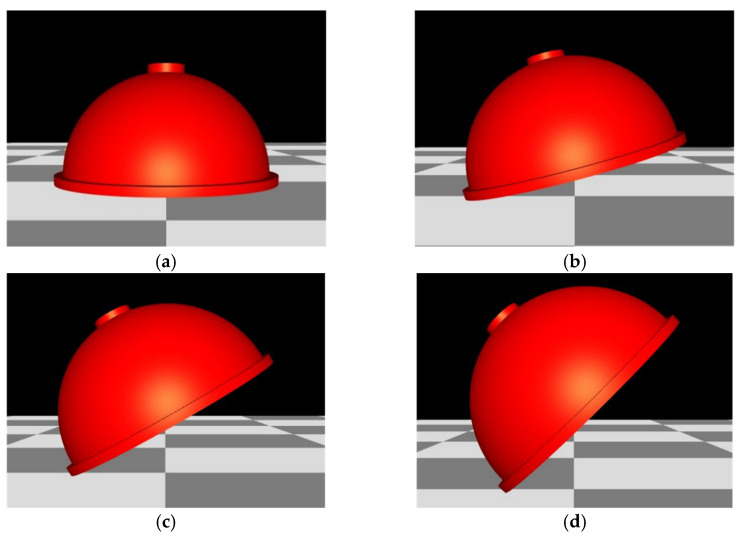
Schematic diagram of different forming angles of the acetabular cup: (**a**) 0°; (**b**) 15°; (**c**) 30°; (**d**) 45°.

Since the parts are prone to warp deformation during SLM forming, the thermal deformation of the acetabular cup at different forming angles (the angle between the acetabular cup and the substrate) was simulated using ANSYS Additive 2019 R3 software, so as to select the best forming angle. When the forming angle exceeds 45°, the overhang angle of the model is too large, and the trabecular coating also needs to be supported, which will affect surface quality. Therefore, the forming angles of 0°, 15°, 30°, and 45° were simulated, as shown in Figure 13.

**Figure 14:**
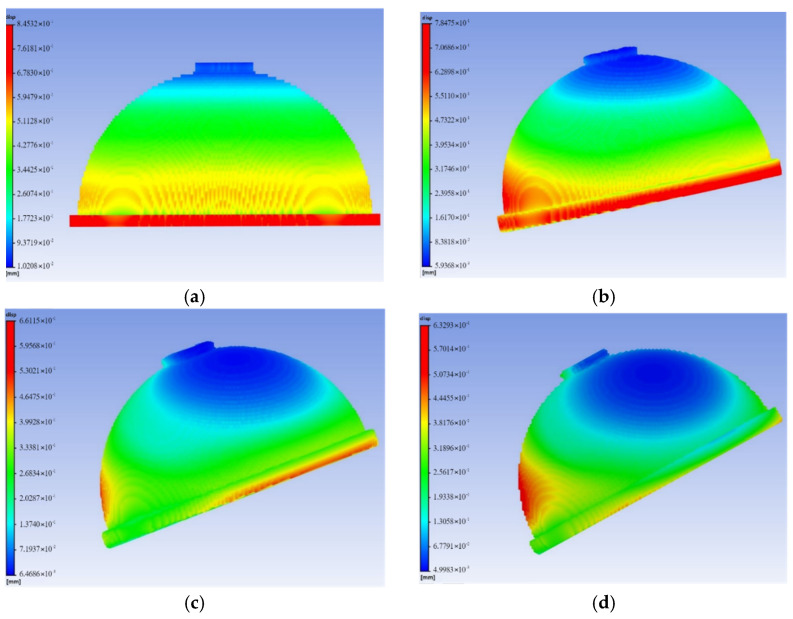
The deformation cloud diagrams of the acetabular cup under different forming angles: (**a**) 0°; (**b**) 15°; (**c**) 30°; (**d**) 45°.

The deformation cloud diagrams of the acetabular cup under different forming angles were shown in Figure 14. It could be seen that when the forming angle is 0°, the maximum deformation is 8.45 × 10^−1^ mm. When the forming angle is 45°, the forming deformation is the smallest, which is 6.33 ×10^−1^ mm. Therefore, the acetabular cup was printed with a forming angle of 45°.

**Figure 15:**
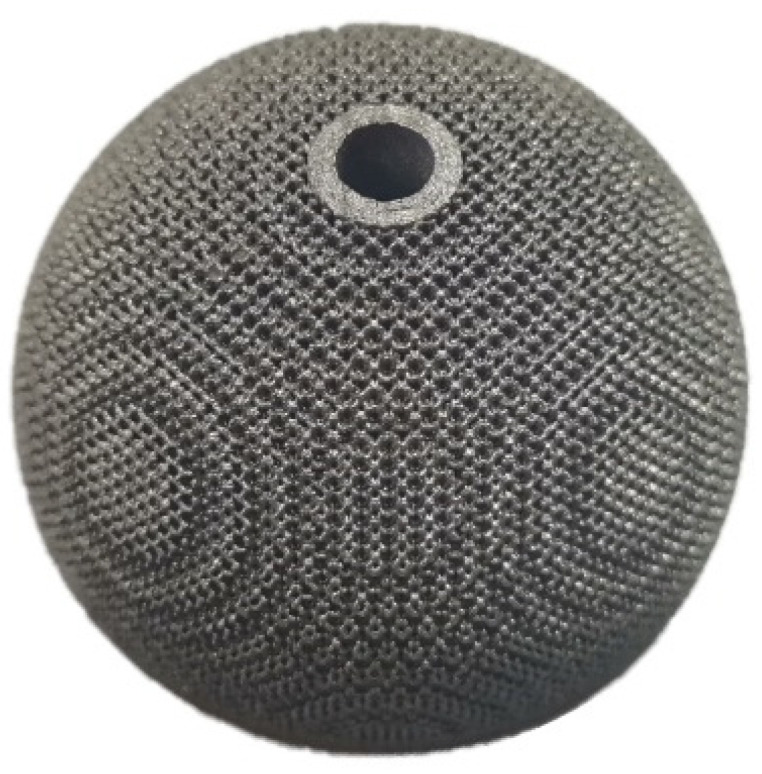
Acetabular cup with a gradient porous structure outer surface formed using SLM.

After the acetabular cup was formed using SLM technology, it needed to be sandblasted and ultrasonically cleaned. The acetabular cup was annealed at 820 °C/2 h to remove residual stress [38], and the acetabular cup with a gradient porous structure outer surface was finally obtained, as shown in Figure 15.

## 4. Conclusions

In view of the stress shielding problem of traditional acetabular cups in joint arthroplasty, we proposed a new design for a trabecular acetabular cup with a gradient porous structure. The following conclusions were obtained:

1. A rhombic dodecahedron porous structure was designed, and the porous structure cells with sizes of 1, 1.5, and 2 mm were compared. By changing the diameter of the struts in the range of 100–300 μm, the porosity was controlled within 60–90%, and the pore size was between 500–1200 μm, which met the requirements for bone ingrowth.
2. Porous Ti6Al4V specimens with cell sizes of 1, 1.5, and 2 mm, and different strut diameters, were printed using SLM technology and tested. Compression test results showed that the specimens with a cell size of 1.5 mm had a compressive strength of 78.16–242.94 MPa and an elastic modulus of 1.74–4.17 GPa, which were close to the values of human cortical bone.
3. Finite element analysis of the hip prosthesis model under different gait conditions was carried out, and the stress map of the trabecular coating on the surface of the acetabular cup was obtained. According to the stress map, the trabecular coating was divided into six stress regions (A–F), and the stress values of each region were determined. The porous Ti6Al4V alloy with the corresponding compressive strength was filled in each area of the trabecular coating, and a trabecular acetabular cup with a porosity of 70.71–89.94% was obtained. This design optimized the porous layer on the outer surface of the traditional titanium alloy acetabular cup and could alleviate the “stress shielding” effect to the greatest extent.
4. The thermal deformation and overhang problems were simulated before printing and the forming angles of 0°, 15°, 30°, and 45° were simulated. The simulation results showed that the forming deformation was the smallest when the forming angle was 45°. Lastly, the acetabular cup was printed by SLM technology, and heat treatment was performed to remove residual stress.

However, this study still has some limitations. Because the electronic universal testing machine can only provide force in a single direction, it cannot simulate the actual force of porous titanium alloy in the hip joint. Therefore, the multi-directional force of the acetabular cup can be studied by simulating the shape of the hip joint and designing corresponding fixtures in future. Alternatively, the elastic constant matrix of the material can be defined in a more accurate way in the finite element analysis, and a porous titanium alloy material representative volume element (3 × 3 × 3) can be used to construct the structure and properties, so as to reduce the analysis time and simulate the stress state of porous titanium alloy more accurate. In addition, in order to ensure that the acetabular cup can be applicated in practice, warping deformation and residual stress in the SLM manufacturing process, as well as the fatigue performance of an acetabular cup with a porous structure, should be focused on in future research.

## Figures and Tables

**Table 1:** Chemical composition of Ti6Al4V ELI powder.

Powder	N	C	H	Fe	O	Al	V	Ti
Ti6Al4V ELI	0.012	0.012	0.0052	0.17	0.12	6.48	3.96	Bal
ASTM F136 Standard	0.05	0.08	0.012	0.25	0.13	5.5–6.5	3.5–4.5	Bal

**Table 2:** Geometric parameters of the rhombic dodecahedron porous structure.

Cell Size, U (mm)	Strut Diameter, S (μm)	Porosity, P (%)	Pore size, A (μm)
1	100	90.59	607.1
	125	85.87	582.1
	150	80.47	557.1
	175	74.55	532.1
	200	68.23	507.1
	225	61.65	482.1
1.5	125	89.94	935.7
	150	85.88	910.7
	175	81.28	885.7
	200	76.21	860.7
	225	70.71	835.7
	250	64.86	810.7
2	150	88.99	1264.2
	175	85.31	1239.2
	200	81.17	1214.2
	225	76.64	1189.2
	250	71.73	1164.2
	275	66.49	1139.2
	300	60.95	1114.2

**Table 3:** Material properties of the total hip joint model.

Model	Material	Elastic Modulus (GPa)	Poisson’s Ratio
Acetabulum	Cortical bone	20	0.3
Liner	UHMWPE	1	0.45
Acetabular cup, femoral head, and stem	Ti6Al4V	113	0.342

**Table 4:** SLM parameters.

Laser Power (W)	Scan Speed (mm/s)	Scan Spacing (μm)	Powder Layer Thickness (μm)
200	1200	140	30

**Table 5:** Stress values in regions A–F and compressive strength and porosity of Ti6Al4V alloy.

Region	Stress (MPa)		Porous Ti6Al4V Alloy	
	Simulated Value	Scaled Value	Compressive Strength (MPa)	Porosity (%)
A	1.827	219.24	221.08	70.71
B	1.402	168.24	185.42	76.21
C	1.130	135.60	154.32	81.28
D	0.857	102.84	118.65	85.88
E	0.584	70.08	78.16	89.94
F	0.312	37.44	78.16	89.94

## Data Availability

Not applicable.
